# Experimental Study on Interface Debonding Defect Detection and Localization in Underwater Grouting Jacket Connections with Surface Wave Measurements

**DOI:** 10.3390/s25113277

**Published:** 2025-05-23

**Authors:** Qian Liu, Bin Xu, Xinhai Zhu, Ronglin Chen, Hanbin Ge

**Affiliations:** 1 College of Civil Engineering, Huaqiao University, Xiamen 361021, China; hqulq@stu.hqu.edu.cn (Q.L.); oscar@hqu.edu.cn (R.C.); 2 Key Laboratory for Intelligent Infrastructure and Monitoring of Fujian Province, Huaqiao University, Xiamen 361021, China; 3 Shanghai Municipal Engineering Design Institute (Group) Co., Ltd., Shanghai 200092, China; 4 Department of Civil Engineering, Meijo University, Nagoya 468-8502, Japan; gehanbin@meijou.ac.jp

**Keywords:** underwater grouting jacket connection (GJC), interface debonding defect, surface wave measurement, defect visualization, experimental study, piezoelectric lead zirconate titanate (PZT), abnormal value analysis

## Abstract

Interface debonding between high-strength grouting materials and the inner surfaces of steel tubes in grouting jacket connections (GJCs), which have been widely employed in offshore wind turbine support structures, negatively affects their mechanical behavior. In this study, an interface debonding defect detection and localization approach for scaled underwater GJC specimens using surface wave measurements with piezoelectric lead zirconate titanate (PZT) actuation and sensing technology was validated experimentally. Firstly, GJC specimens with artificially mimicked interface debonding defects of varying dimensions were designed and fabricated in the lab, and the specimens were immersed in water to replicate the actual underwater working environment of GJCs in offshore wind turbine structures. Secondly, to verify the feasibility of the proposed interface debonding detection approach using surface wave measurements, the influence of the height and circumferential dimension of the debonding defects on the output voltage signal of PZT sensors was systematically studied experimentally using a one pitch and one catch (OPOC) configuration. Thirdly, a one pitch and multiple catch (OPMC) configuration was further employed to localize and visualize the debonding defect regions. An abnormal value analysis was carried out on the amplitude of the output voltage signals from PZT sensors with identical wave traveling paths, and the corresponding abnormal surface wave propagation paths were identified. Finally, based on the influence of interface debonding on the surface wave measurements mentioned above, the mimicked interface debonding defect was detected successfully and the region of debonding was determined with the intersection of the identified abnormal wave travelling paths. The results showed that the mimicked debonding defect can be visualized. The feasibility of this method for interface debonding defect detection in underwater GJCs was confirmed experimentally. The proposed approach provides a novel non-destructive debonding defect detection approach for the GJCs in offshore wind turbine structures.

## 1. Introduction

Grouted jacket connections (GJCs), comprising a pile guide jacket, steel tube pile, and interposed grouting material, represent one of the most prevalent structural interfaces between offshore wind turbine pile guide jackets and steel pile foundations. As critical components of offshore wind support structures, GJCs rely on the integrity of the high-strength grouting material–steel tube interface to ensure structural safety and serviceability under harsh environmental conditions and dynamic loading [1,2,3]. In steel tube–cementitious material composite structures, such as concrete-filled steel tube (CFST) and grouted connections, interface debonding defects disrupt the shear transfer and deformation compatibility between the steel tube and core material, leading to degradation of the structural and mechanical performance [4,5]. Interface debonding between grouting materials and steel tubes is a common issue observed in offshore wind turbine support systems. Such defects can potentially cause slippage between grouting materials and steel tube piles and increase the risk of complete system collapse [6,7,8,9]. The accurate identification of internal interface debonding defect in GJCs for offshore wind turbine supports as an inaccessible defect remains a challenging task due to the shielding effects of outer steel tubes and underwater operational conditions. The traditional non-destructive testing techniques for reinforced concrete (RC), steel structures, and concrete–steel composite structures often show limited effectiveness in such scenarios. There is a critical and pressing demand in the development of advanced techniques to reliably detect and visualize inaccessible interface debonding defects in GJCs for offshore wind turbine infrastructure.

In recent years, piezoelectric lead zirconate titanate (PZT) patches have been utilized as actuators and sensors to detect defects in different engineering structures such as RC structures, steel structures, concrete–steel composite structures, and carbon fiber-reinforced polymer (CFRP) structures. Leveraging both the actuating and sensing functions of PZT materials, researchers have developed PZT aggregates that are embeddable in concrete to monitor the material performance and detect defects such as cracking, interface debonding in CFRP structures, and bond–slip defects between steel tubes and concrete in concrete–steel composite structures [10,11,12]. As a local defect detection method, electromechanical impedance (EMI) measurement from embedded or surface-mounted PZT sensors can identify defects within a certain sensitivity range of the PZT sensors, enabling defect localization. Based on EMI measurements from surface-mounted PZT patches, Liu et al. [13] investigated the sensitivity range of interface debonding defects in concrete-filled steel tube (CFST) members and successfully located interface debonding defects in practical engineering structures in a non-destructive way. Moll [14] successfully identified 4 mm void defects in grouted connection models using the EMI measurement method. However, a limitation of this method is that the EMI measurements from PZT sensors are only sensitive to local defects. To detect interface debonding in a real engineering structure, densely distributed PZT sensors are required. In contrast, stress wave measurement methods are more applicable for inspecting large-scale engineering structures because defects along the wave travelling path lead to variations in stress wave propagation and PZT sensor measurements.

Based on stress wave measurements using PZT patches, Zhang et al. [15] monitored damage in L-shaped CFST columns under low cyclic loading, while Kocherla et al. [16] achieved concrete crack monitoring with embedded PZT sensors. Zhang et al. [17] employed the piezoelectric stress waves method to effectively monitor the loosening process of a wedge anchorage system and utilized wavelet package energy (WPE) to quantitatively evaluate the loosening of steel strands. Tareen et al. [18] monitored the interface debonding defects in irregular multi-cavity steel reinforced concrete (SRC) columns using PZT sensors. Xu et al. [19] proposed an interface debonding defect detection approach for CFST structures using bulk stress wave measurements and numerically investigated the mechanisms. To explore the effect of mesoscale concrete heterogeneity in CFST components on bulk wave propagation, Wang et al. [20] established a substructure mesoscale model and analyzed the wave characteristics numerically. The results showed that the impact of the interface debonding defects on the response of PZT sensors is dominant compared to that of the heterogeneous mesoscale structure of the concrete in CFST members.

Due to the obvious attenuation of bulk waves in mass concrete in large-scale CFST members, interface debonding defect detection for large-scale CFST members using bulk stress waves is particularly challenging. In recent years, surface wave measurement approaches using surface-mounted PZT sensors have received more attention, and their advantages in non-destructive testing for various engineering structures have been widely recognized [21,22,23]. Lu et al. [24] conducted experimental investigations on stress wave propagation characteristics in rebar-reinforced concrete beams, demonstrating the feasibility of stress wave-based techniques for structural damage identification through a waveform analysis. Li et al. [25] proposed a damage identification method for plate structures based on PZT patch arrays, enabling the quantitative imaging of varying degrees of damage by accounting for the attenuation of Lamb wave propagation in plate structures. Chen et al. [26] further numerically analyzed the mechanism of the interface debonding detection approach for CFST members using surface wave measurements. The results showed that interface debonding defects significantly reduce the wave energy transmission from steel tubes to the core concrete, and the responses and WPE values of surface-mounted PZT sensors of the corresponding surface wave travelling path with interface debonding defects are higher than those of the surface wave travelling path without such interface debonding defects. Cui et al. [27] combined piezoelectric surface wave measurements with data-driven deep learning (DL) to achieve damage imaging in the skin, flange, and wing cover areas of reinforced composite structural panels. Liu et al. [28] developed a hybrid approach combining surface wave and EMI measurements, enabling the rapid detection of both artificially mimicked and naturally occurring interface debonding defects in CFST specimens. Zhu et al. [29] numerically and experimentally verified the feasibility of the interface debonding defect detection method in GJCs based on surface wave measurements from surface-mounted PZT sensors. The findings indicated that this approach can effectively detect interface debonding defects in GJCs with a planner dimension of 50 mm, and the detected localization of these defects met the real localization of the mimicked defects in specimens. Given that some GJCs in offshore wind turbines are submerged in seawater and seawater affects stress wave propagation along the steel tube, Xu et al. [30] established a finite element model for underwater GJCs. By analyzing stress wave fields, they revealed the underlying detection principles of surface wave measurements and validated the effectiveness of this approach. However, regarding the feasibility of using surface wave detection to identify interface debonding defects in underwater GJCs of offshore wind support structures, further experimental research is still required to thoroughly investigate this method.

In this study, aiming at the detection and localization of interface debonding in underwater GJCs of offshore wind turbine support structures, a method based on surface wave measurements using PZT active actuating and sensing technology was proposed and experimentally investigated. Two GJC specimens with interface debonding defects of varying dimensions were designed and fabricated in the lab. After installing the PZT actuator and sensors on the outer surface of the outer steel tube, the specimens were immersed in water for the surface wave measurements. Firstly, the surface wave measurements were carried out in a one pitch and one catch (OPOC) configuration, and the influences of the existence of interface debonding defects and dimensional variations on the output voltage signal and wavelet packet energy (WPE) value of the surface-mounted PZT sensors were analyzed, thereby validating the feasibility of the proposed interface debonding detection method. Based on the above findings, to comprehensively detect and localize the interface bonding conditions between the outer steel tube and grouting material in GJC components, a one pitch and multiple catch (OPMC) configuration was further employed. Through an analysis of the abnormal values in the surface wave measurements from the PZT sensors in each group, with an identical surface wave travelling direction and length, the surface wave travelling paths with interface debonding defects were successfully identified. Based on the above found relation between the interface debonding defects and the corresponding surface wave measurements, an interface debonding region estimation approach using the intersection of the identified surface wave travelling paths with abnormal surface wave measurements was proposed, which enables the visualization of interface debonding defect regions for underwater GJCs. The experimental results demonstrated that the proposed method can successfully detect the existence of interface debonding defects in underwater GJC specimens and estimate the localization of the mimicked interface debonding defects within acceptable accuracy.

## 2. Principle of Interface Debonding Detection Based on Surface Wave Measurements for Underwater GJCs

In this study, an innovative non-destructive testing method for detecting interface debonding defects in underwater GJC components using PZT-based stress wave detection technology with PZT actuating and sensing technology was experimentally verified. The developed method employs PZT patches attached to the outer surfaces of the GJC components as both actuators and sensors to generate and collect surface wave signals. When a voltage signal is applied to the PZT actuator, both surface waves (Rayleigh waves) and longitudinal waves (P waves) initiate and propagate in the GJC components due to the converse piezoelectric effect of the PZT material. The surface waves propagating along the steel tube are measured by the PZT sensor, which converts the surface wave travelling from the PZT actuator into electrical signals through the direct piezoelectric effect. Research has shown that surface waves account for 70% of the total wave energy, making them the most significant component and highly sensitive to interface debonding defects along the surface wave propagation paths [26,31]. To experimentally investigate and verify the applicability of the non-destructive testing technology, the use of surface wave measurements for interface debonding defects in underwater GJC components was the major task of this study.

**Figure 1:**
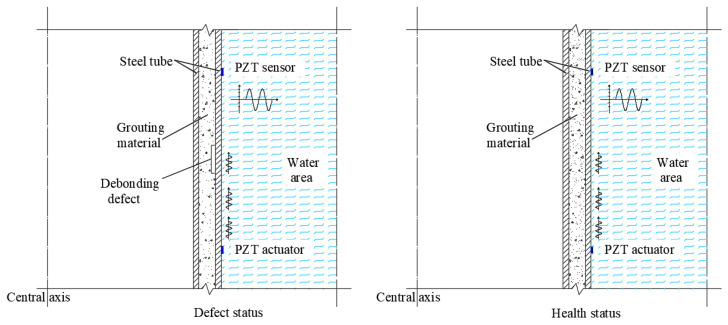
Principle of interface debonding detection of underwater GJCs based on surface wave measurements.

For interface debonding detection in underwater GJC members, PZT patches are strategically installed on the outer surface of the steel tube of the investigated GJC to facilitate its implementation, as schematically illustrated in Figure 1. The PZT actuator is excited by a high-frequency voltage signal, generating stress waves that propagate through both the grouting material and the steel tube of the underwater GJC specimen. When interface debonding defects initiate between the grouting material and the outer steel tube, the energy propagation pattern of the stress waves transitioning from the steel tube to the grouting material differs significantly from that of a GJC specimen without interface debonding. Therefore, by comparing surface wave measurements from PZT sensors along traveling paths with identical distances and orientations, interface defects can be detected through the deviations in surface wave measurements.

## 3. Experimental Study on the Feasibility of the Interface Debonding Detection Approach with Surface Wave Measurements of Underwater GJCs

### 3.1. Design of Scaled Underwater GJC Specimens with Mimicked Debonding Defects

**Figure 2:**
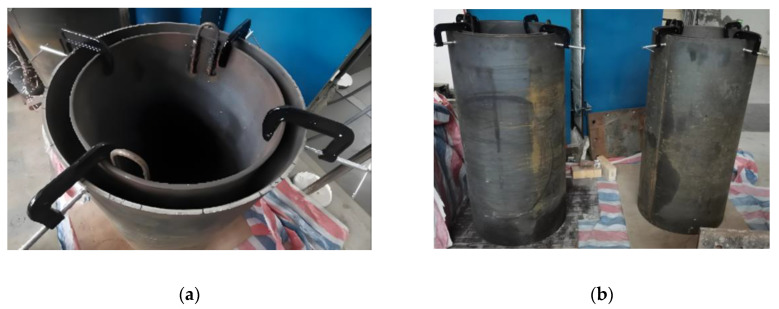
GJC specimens with mimicked defects: (**a**) the two steel tubes being assembled before pouring the grouting; (**b**) the GJCs specimens after pouring the grouting.

To further experimentally verify the detectability of interfacedebonding defects in underwater GJCs using surface wave measurement techniques, two scaled underwater GJC specimens with mimicked interface debonding defects were designed and fabricated in the laboratory. As illustrated in Figure 2, both specimens share an identical height of 1200 mm, with steel tube thicknesses of 10 mm. The inner and outer steel tubes had diameters of 500 mm and 600 mm, respectively, and were made of Q235 steel. A high-strength non-shrinkage grouting material with a concrete strength grade of C60 was injected between the inner and outer steel tubes.

**Figure 3:**
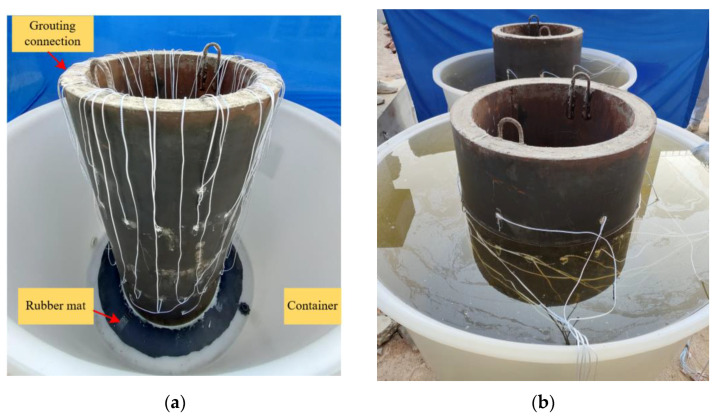
Underwater GJC specimens with mimicked interface debonding defects: (**a**) before water storage of the specimen; (**b**) after water storage of the specimen.

To simulate the actual operation environment of underwater GJCs of offshore wind turbine structures, the GJC specimens were placed in water when each test was carried out. As shown in Figure 3a, a rubber mat was bonded to the bottom of each specimen using an epoxy adhesive to prevent water ingress into the inner steel tube. After the epoxy resin had cured, the container was filled with water to simulate the underwater operation environment, as shown in Figure 3b.

### 3.2. Design and Arrangement of Mimicked Interface Debonding Defects

**Figure 4:**
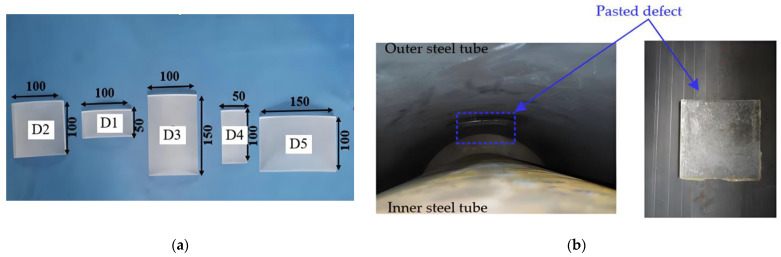
Mimicked interface debonding defects in GJC specimens: (**a**) artificially mimicked defects with different dimensions; (**b**) mimicked interface debonding defects installed on the inner surface of a steel tube (unit: mm).

As illustrated in Figure 4, interface debonding defects at predetermined locations on the inner wall of the outer steel tube were mimicked using acrylic materials, which were bonded with an epoxy resin adhesive prior to the assembly of the inner and outer tubes. A total of five interface debonding defects with different dimensions, numbered D1–D5, were mimicked in the two scaled specimens. Interface debonding defects with varying vertical heights and circumferential lengths were designed, and the OPOC measurement configuration was primarily employed to investigate the influence of different interface debonding defect sizes on the surface wave measurement signal amplitude.

**Figure 5:**
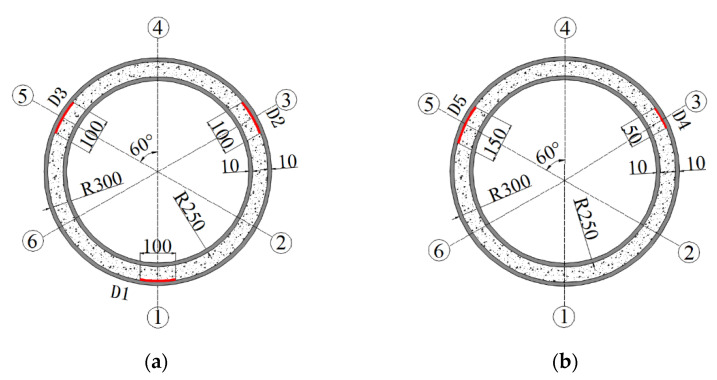
Locations and dimensions of all mimicked interface debonding defects in both specimens: (**a**) specimen A; (**b**) specimen B (unit: mm).

**Figure 6:**
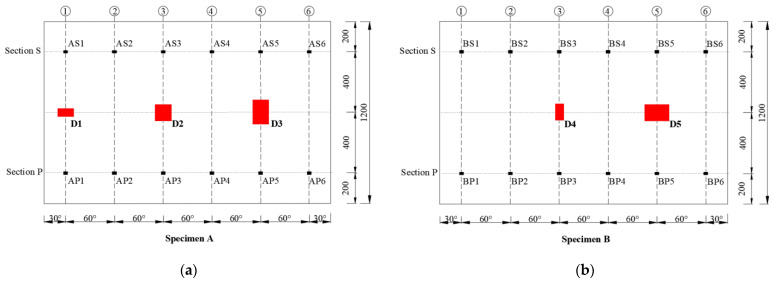
Locations of PZT patches for the OPOC configuration in the net of the lateral surface of each specimen: (**a**) specimen A; (**b**) specimen B (unit: mm).

Interface debonding defects D1, D2, and D3 were installed in scaled GJC specimen A, and defects D4 and D5 were installed in scaled GJC specimen B. The detailed dimensions of the mimicked debonding defects can be found in Table 1. The centers of each mimicked defect were 600 mm from the bottom surfaces of the two specimens. The locations and dimensions of all mimicked interface debonding defects in both specimens are shown in Figure 5, where six generatrices are uniformly distributed with an identical central angle of 60-degree. The installation locations of all mimicked debonding defects in the net of the lateral surface of each specimenare illustrated in Figure 6.

### 3.3. OPOC Configuration for Surface Stress Wave Measurements

To experimentally verify the feasibility and accuracy of the proposed method for interface debonding defect detection and localization, both the one pitch and one catch (OPOC) and one pitch and multiple catches (OPMC) surface wave measurement configurations were employed in the test. Table 1 shows the measurement paths corresponding to each mimicked interface debonding defect. Each PZT patch was made of PZT-5A material, with both electrodes located on the same side. The dimensions were 15 mm × 10 mm × 0.3 mm.

In surface wave measurements using the OPOC configuration, Figure 6 illustrates the positions and numbering of both the mimicked defects and surface-mounted PZT patches in the expanded side view of the outer steel tube for the two GJC specimens. The PZT patches are installed on the outer steel tube at two cross-sections of each specimen. Six PZT sensors are set on Section P, which is 200 mm from the bottom of the specimen, and are named as P1–P6. Six PZT actuators are set on Section S, which is 1000 mm from the bottom of the specimen, and are named as S1–S6.

**Figure 7:**
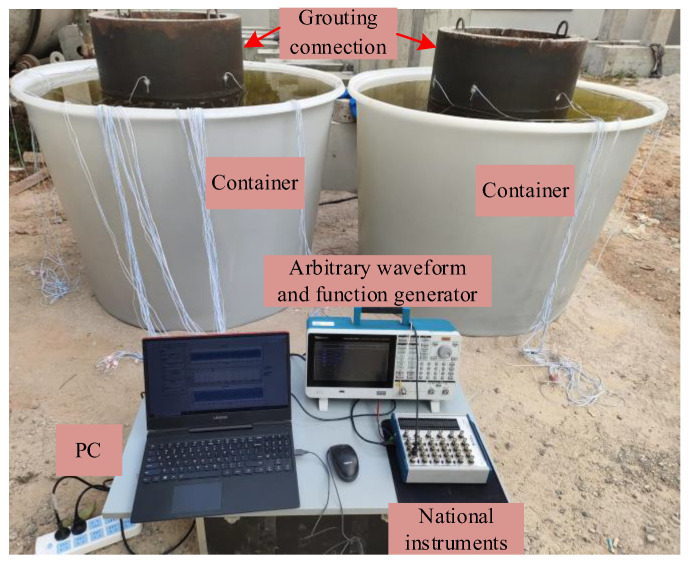
Test setup for surface wave measurement for GJC specimens.

The surface wave measurement system employed in this experimental study consists of an arbitrary waveform and function generator, a data acquisition system for measuring PZT sensor responses, and a laptop workstation for data storage and analysis. Figure 7 illustrates the measurement system and GJC specimens with PZT actuators and sensors. The frequency selection of the excitation signal critically impacts the accuracy of the defect identification. Chen et al. and Xu et al. provided frequency selection criteria for interface debonding detection [26,32]. At lower excitation frequencies, the longer wavelength of the stress waves reduces the spatial resolution, leading to undetected minor defects. At higher excitation frequencies, the stress wave energy dissipates more rapidly, leading to suboptimal interface debonding detection outcomes. Considering the propagation mechanisms and attenuation characteristics of stress waves within GJC components, a five-cycle, 20 kHz sinusoidal windowed signal was directly applied to the surface-mounted PZT actuators.

### 3.4. Interface Debonding Defect Identification Results Based on Surface Wave Measurements with an OPOC Configuration

#### 3.4.1. Effect of Interface Debonding Defects Height on PZT Sensor Measurements

**Figure 8:**
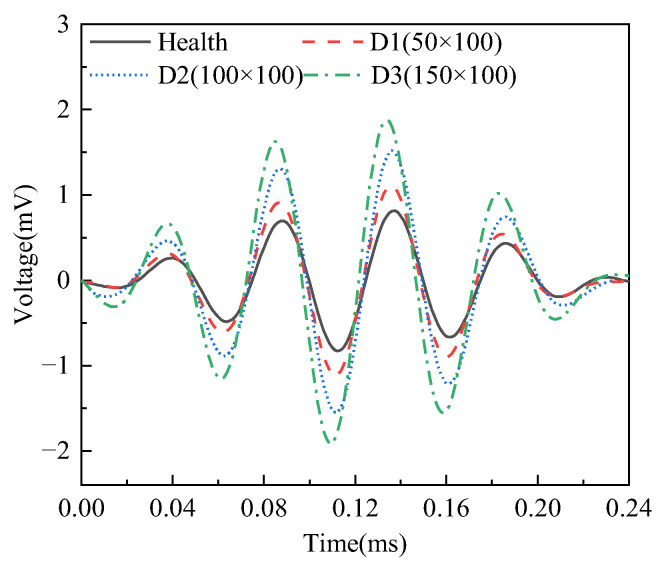
Time domain signal of output voltage of surface wave measurements from surface-mounted PZT sensors corresponding to interface debonding defects with different heights.

The responses of PZT sensors in different surface wave travelling paths with and without mimicked interface debonding defects in specimen A are exhibited in Figure 8. It can be observed from Figure 8 that the amplitude of the measured surface wave signal from the surface-mounted PZT sensor corresponding to the healthy travelling path (AS2–AP2) is the smallest, while the surface wave signals from the PZT sensors corresponding to the travelling path with mimicked interface debonding defects exhibit significantly higher amplitudes. The test finding validates that the presence of an interface debonding defect that reduces the propagation of the stress wave into the grouting material, retaining more wave energy in the steel tube and travelling as surface waves along the steel tube. Moreover, when the radial and circumferential dimension of the mimicked interface defect are identical, the amplitudes of the PZT sensors corresponding to surface travelling paths AS1–AP1, AS3–AP3, and AS5–AP5 increase significantly with the increase in height of the mimicked debonding defects, as shown in Figure 8.

**Figure 9:**
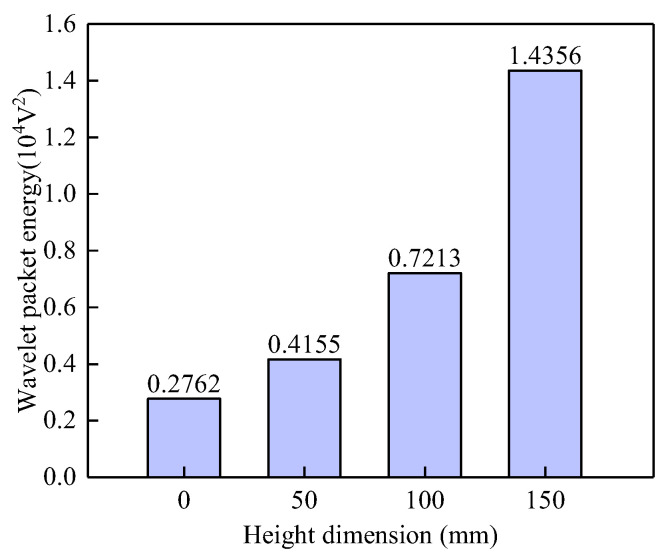
Wavelet packet energy of the surface wave measurements from surface-mounted PZT sensors corresponding to interface debonding defects with different heights.

To intuitively identify the relationship between the vertical height of the interface debonding defect and the PZT output voltage signal, the WPE values of the PZT sensor measurements were calculated under different bonding conditions. As shown in Figure 9, the WPE value of the output signal from the PZT sensor in a healthy state is the lowest. When an interface debonding defect initiates along the surface wave propagation path, the stress wave transmitted into the grouting material during propagation decreases, resulting in an increase in the energy received by the surface-mounted PZT sensor. When the debonding defect exists, there is a significant positive correlation between the WPE value and the height of the debonding defect.

#### 3.4.2. Effect of Circumferential Dimension of Interface Debonding Defects on PZT Sensor Surface Wave Measurements

**Figure 10:**
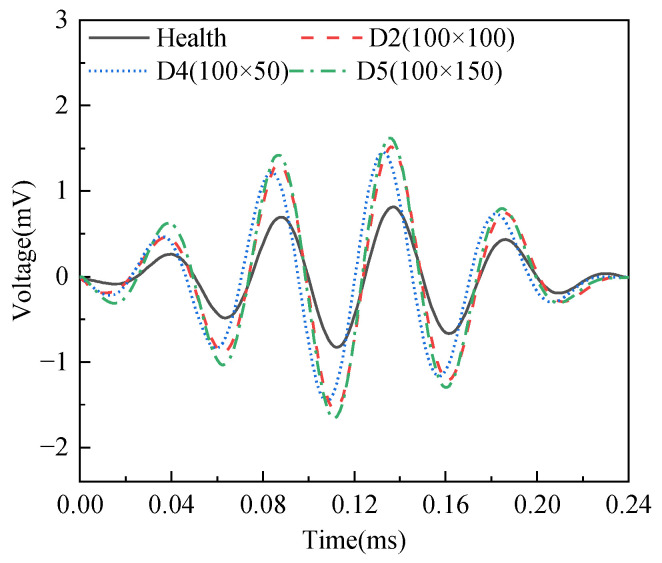
Time domain signal of surface wave measurements from surface-mounted PZT sensors corresponding to interface debonding defects with different circumferential dimensions from both specimens A and B.

The responses of the surface-mounted PZT sensors along the surface wave travelling paths with mimicked interface debonding of different circumferential dimensions but an identical height of 100 mm in both specimens A and B are exhibited in Figure 10. Consistent with the findings presented in Figure 8, the amplitudes of the signals from surface-mounted PZT sensors located along the surface wave travelling path with interface debonding defects, including BS3–BP3, AS3–AP3, and BS5–BP5, are significantly larger than those of the surface-mounted PZT sensors along the surface wave travelling paths without a defect, such as BS4–BP4. However, as the circumferential dimension of the debonding defects changes, the differences in the amplitudes of the PZT sensor responses are relatively small but still detectable. This finding indicates that the responses of PZT sensors are less sensitive to variation in the circumferential dimension of the debonding defect, when it exceeds 50mm and the OPOC configuration is used for the surface wave measurement.

**Figure 11:**
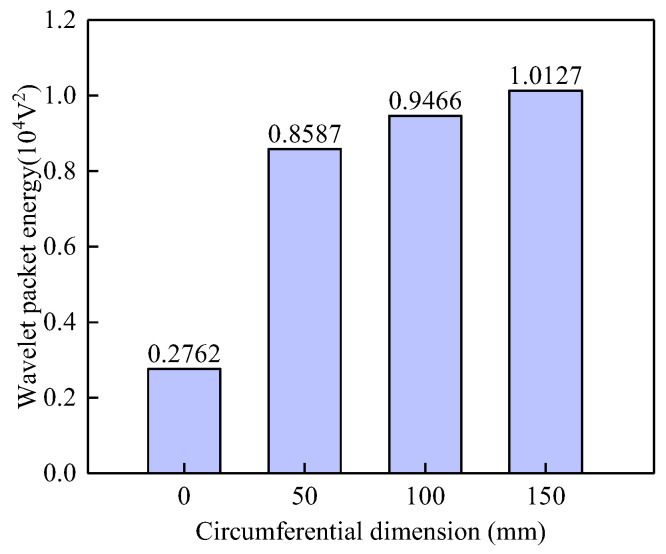
Wavelet packet energy of PZT sensors corresponding to surface wave propagation paths with different circumferential dimensions in both specimens A and B.

Figure 11 presents the WPE values of the surface-mounted PZT sensor measurements along the surface wave traveling paths with interface debonding defects of varying circumferential dimensions but an identical height. The results indicate that the WPE values of the PZT sensors along the surface wave propagation paths with interface debonding defects show no significant variation, even though the circumferential defect dimensions are different. An interface debonding with a circumferential dimension of 50mm leads to an obvious increase in the WPE value of surface wave measurement when compared with that of surface wave measurement of a travelling path without an interface debonding. This result conclusively validates the sensitivity and feasibility of surface wave measurement-based interface debonding defects detection methods for underwater GJCs.

## 4. Experimental Verification on Localization Approach of Interface Debonding in Underwater GJC Specimens

### 4.1. OPMC Configuration for Interface Debonding Localization Using Surface Wave Measurements

In practice, the actual regions of interface debonding defects in GJC specimens are unknown before testing. It is essential to develop a method for identifying and localizing the debonding regions in GJCs using surface wave propagation measurements. To address this, a OPMC surface wave measurement configuration was proposed and experimentally verified to identify and visualize the regions of interface debonding defects in GJCs.

**Figure 12:**
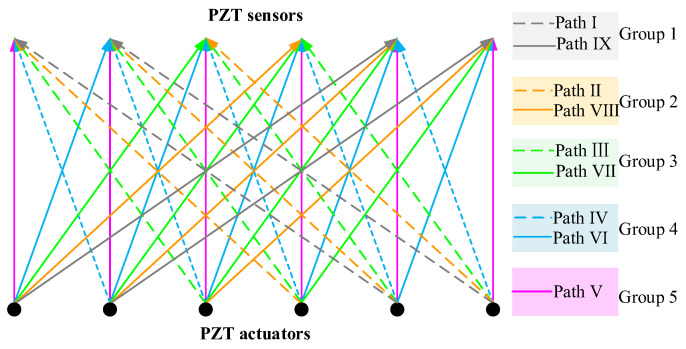
OPMC measurement configuration and path definition.

In the OPMC configuration, one surface-mounted PZT actuator is used as an actuator and excited by an excitation voltage signal, and nine surface-mounted PZT sensors are employed as sensors to measure the surface waves propagating along different travelling paths. The surface wave travelling paths with identical stress wave travelling lengths and orientations are grouped for comparison and displayed with an identical color, as shown in Figure 12. In a one pitch and nine catches configuration, there are a total of nine surface wave propagating paths for each PZT actuator, and the surface wave propagating paths can be grouped into a total of five groups (groups 1 to 5). It is clear that the surface wave measurements of the PZT sensors in each group will be close if no interface debonding defects exist in each path and in each group. Because interface debonding defects lead to surface wave measurement increases of the corresponding surface wave propagating path, abnormal analyses on surface wave measurements can be performed on each group to identify the abnormal surface wave traveling paths.

### 4.2. Arrangement of Surface-Mounted PZT with a OPMC Configuration for Interface Debonding Defect Localization

**Figure 13:**
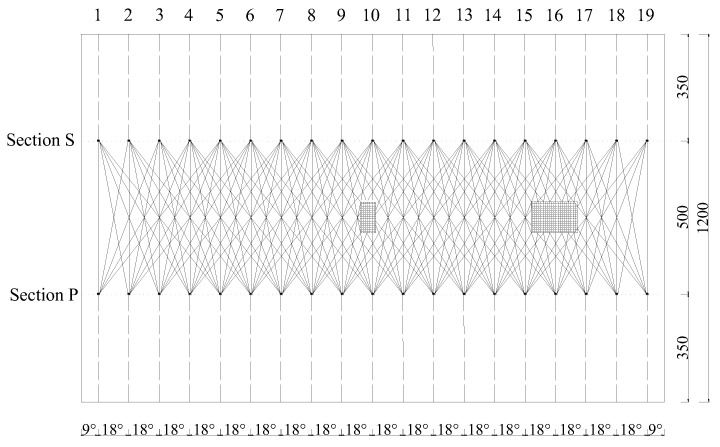
Locations of PZT patches for the OPMC configuration in the net of the lateral surface of specimen B (unit: mm).

To further experimentally verify the feasibility of the proposed interface debonding defect localization approach, an OPMC configuration was employed to measure the surface waves of multiple travelling paths of the specimens. Figure 13 shows the OPMC configuration of the PZT actuator and sensor arrangements for underwater GJC specimen B. All PZT patches are localized at two different cross-sections called sections S and P, as shown in Figure 13. The distance in the vertical direction of the two cross-sections is 500 mm. The PZT patches are arranged at intervals of 100 mm along the circumferential direction of the specimen on sections S and P as actuators and sensors, respectively. Section S is located 850 mm from the bottom, while section P is 350 mm from the bottom. The actuators on section P are labeled P1–P19, while the sensors on section S are labeled S1–S19. It is worth noting that after the installation of the PZT sensors and actuators, a waterproof layer should be applied to the PZT surfaces and the welding joints of the dual-core shielded wire to prevent electrical leakage.

### 4.3. Estimation of Interface Debonding Region Based on OPMC Measurement Configuration

Taking specimen B as an example, PZT patches are circumferentially spaced at 100 mm intervals on the outer lateral surface of the steel tube of the specimen. Nineteen PZT actuators (S1–S19) and nineteen PZT sensors (P1–P19) are arranged on sections S and P, respectively. The positions of the surface-mounted PZT patches in the net of the lateral surface of the steel tube are shown in Figure 13. During testing, a continuous sinusoidal signal with a frequency of 20 kHz and an amplitude of 10 V was employed to excite each PZT actuator. Surface wave measurements were made using the OPMC configuration. When one PZT was used as an actuator, the responses of 9 PZT sensors corresponding 9 surface wave travelling paths were recorded. Therefore, a total of 171 measurements were recorded. All surface wave travelling paths were grouped into 5 groups.

Therefore, an abnormal value analysis on the amplitude of the surface wave measurements in each group was carried out following the criteria outlined in the Technical Specification for Inspection of Concrete Defects by Ultrasonic Method (CECS 21-2000) [33]. As interface debonding defect leads to an increase in the surface wave measurements, unlike the abnormal values judgment method described in the [33], where smaller values are treated as abnormal values, in this study, the values that are larger than the determined abnormal threshold are treated as abnormal values, and the corresponding surface wave travelling paths can be treated as the travelling paths with interface debonding defects.

Using a one pitch and nine catches configuration, surface wave propagation paths with identical stress wave travelling distances and directions are grouped into identical groups, and an abnormal value analysis of the amplitudes of the surface wave measurements in each group is carried out. Subsequently, the judgement value of the amplitudes for each group are determined. By comparing and analyzing time domain signals of each path within each group, the abnormal paths are preliminarily identified. Paths with amplitudes greater than the judgement value are identified as abnormal, signifying debonding defects along those trajectories.

In each group, the amplitude derived from the surface wave measurements were sorted in ascending order, represented as *X*_1_ ≤ *X*_2_ ≤ ... ≤ *X_n_* ≤ *X_n+_*_1_, where *n* + 1 is the total number of surface wave measurements in each group. A significantly larger data point than the adjacent one in each group is judged as suspicious data. The smallest candidate suspicious data point (denoted as *X_n_*) and its preceding data points are subjected to a statistical analysis. The arithmetic mean (*m_x_*) and standard deviation (*s_x_*) are calculated by the following Equations (1) and (2), respectively. These parameters are subsequently substituted into Equation (3) to derive the judgment value *X*_0_, which serves as the discriminant boundary for abnormal data classification.

(1) $$m_{x}=\Sigma X_{i}/n$$

(2) $$s_{x}=\sqrt{\left(\Sigma X_{i}^{2}-n\cdot m_{x}^{2})/(n-1\right)}$$

(3) $$X_{0}=m_{x}+\lambda_{1}\cdot s_{x}$$

where *λ*_1_ is derived from the specification in [33], *X_i_* represents the *i*-th measurement value, and *n* denotes the total number of data included in the statistical analysis.

The judgment value *X*_0_ is compared with the minimum value *X_n_* of the suspicious data. If *X*_0_ ≤ *X_n_*, *X_n_* and all subsequent data points (*X_n_*, *X_n_*_+1_, ...) are classified as abnormal values and removed. The remaining dataset (*X*_1_~*X_n−_*_1_) is then iteratively reanalyzed using Equations (1)–(3) until no further abnormal values are detected. Conversely, if *X*_0_ > *X_n_*, the next ordered value *X_n_*_+1_ should be included in the calculation and re-evaluation process.

Table 2 indicates the amplitudes of output voltage signals from PZT sensors corresponding to each surface wave propagation path of specimen B. Based on the abnormal value analysis method described above, a judgment value was calculated for each group. In each group, measurement amplitudes greater than the judgment value are highlighted in red bold font, indicating that the corresponding surface wave propagation paths contain interface debonding defects.

**Figure 14:**
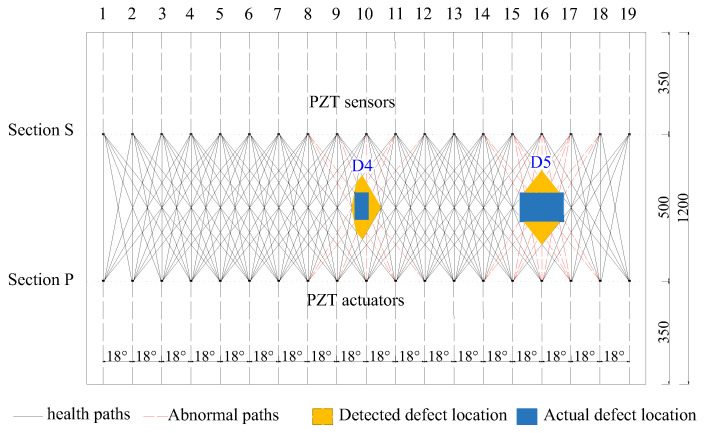
Abnormal paths and detected interface debonding defect regions in the net lateral surface of specimen B (unit: mm).

The surface wave propagation paths corresponding to the identified abnormal amplitudes are highlighted in red in Figure 14. Based on the experimentally verified relationship between interface debonding and the corresponding surface wave measurements of a PZT sensor, the region of interface debonding in the lateral surface of specimen B was estimated by the intersection of the identified abnormal surface wave travelling paths in each group. Based on the identified abnormal surface wave travelling paths corresponding the abnormal values shown in Table 2, two interface debonding defect regions in lateral surface of specimen B were estimated, as shown in Figure 14, which can be treated as a visualization of the two mimicked interface debonding defects of D4 and D5 in specimen B. It is worth noting that the proposed approach identified the locations of the two mimicked interface debonding with acceptable accuracy, even though the estimated regions of the mimicked interface debonding defects were greater than the actual ones.

### 4.4. Discussion on the Detection and Localization Results for Interface Debonding Defects

A non-destructive testing method for identifying and locating interface debonding defects in GJC components has been proposed by combining OPMC surface wave measurements with an abnormal analysis, based on surface-mounted PZT patches. To evaluate the effectiveness of the multi-defect identification, localization, and visualization method, two interface debonding defects of different sizes were introduced into the GJC specimen. As shown in Figure 14, the center of each identified interface debonding defect region shown in yellow matches well with the actually mimicked interface debonding defect region shown in blue, validating the precise positioning capability of the detection and localization approach using OPMC surface wave measurements. In the detection of Defect D5, the center of the identified defect area coincides perfectly with the actual defect. Even a 16 mm difference was observed between the center of the identified defect region and the actual defect region of D4, it is acceptable is engineering practice. Moreover, although the identified interface debonding regions corresponding to the two mimicked defects were greater than the actual debonding area, the results are acceptable from the point of view of engineering application considering the dimensions of the mimicked interface debonding defect are relatively small when compared with the lateral surface of the specimens. As the debonding region is estimated by the identified abnormal surface wave travelling paths, the detectability of interface debonding defects is related to the lateral spacing between the PZT patches, it is understandable that smaller spacing between the PZT patches will be helpful in enhancing the localization accuracy. Moreover, to achieve more precise visualization of interface debonding defects in practical engineering applications, PZT patches could be densely arranged on both the left and right sides of the identified region to make the surface wave measurements.

## 5. Conclusions

This paper presents an experimental verification of an approach for detecting and locating interface debonding defects in underwater GJCs, which are widely employed in offshore wind turbine support structures, based on surface wave measurements from surface-mounted PZT sensors installed with an OPMC configuration and abnormal value analysis. The experimental results, based on an analysis of the PZT sensor measurements of surface wave travelling paths with identical travelling length and orientation, show that the existence and region of the mimicked interface debonding defects in the specimens were successfully detected. The following conclusions can be drawn from this experimental study:

(1) When a mimicked interface debonding defect is located along the surface wave travelling path of an underwater GJC specimen, the experimental results demonstrate that the signal amplitude from the PZT sensors along a surface wave travelling path with interface debonding defects exceeds those along paths without an interface debonding defect under the OPOC configuration. The reason behind this finding is that the existence of interface debonding defects reduces the surface wave propagation from the steel tube to the grouting material, so more stress waves propagate along the steel tube as surface waves. Additionally, as the length of the interface debonding defects in the direction of the wave propagation increases, the measured signal amplitude increases proportionally.
(2) When the interface debonding defect is located on the surface wave travelling path, variations in the width of the interface debonding defect exceeding 50 mm in the circumferential direction have no significant effect on the PZT sensor signals under the OPOC configuration. However, compared to signals measured along healthy surface wave traveling paths, the responses from PZT sensors corresponding to surface wave travelling paths with interface debonding defects increase significantly. The interface debonding detection approach for underwater GJCs is sensitive to the existence of interface debonding defects.
(3) The feasibility of the approach for estimating the distribution region of the interface debonding defects in underwater GJCs using the OPMC configuration and the abnormal value analysis for the surface wave travelling path detection was experimentally verified. Abnormal amplitude values of the PZT sensor measurements in each group with identical surface wave travelling path lengths and orientations were determined. The region of the interface debonding defects can be estimated as the intersection regions covered by the abnormal surface wave travelling paths, which provides a way to visualize the interface debonding regions in underwater GJCs of offshore wind turbine support structures.

This study was an experimental investigation on the feasibility of this interface debonding detection and localization approach for underwater GJCs using surface wave measurements from surface-mounted PZT sensors with an OPMC configuration. As shown in Figure 14, even with the rough location and region identified by the proposed approach, further investigation is desired to improve the detection accuracy to decrease the difference between the identified region and the actual debonding defects. Some local detection approaches, as well as different imaging techniques such as probability-based imaging or tomographic imaging, might be involved after detecting the rough debonding regions for visualization. Additionally, since this study was primarily conducted under laboratory conditions with a simulated static water environment, future work should also consider real engineering applications for full-scale GJCs of offshore wind turbine support structures.

## Figures and Tables

**Table 1:** Interface debonding defect and PZT actuator and sensor configuration.

Specimen Number	Actuator-Sensor	Defect Number and Dimension (Height × Circumferential)
Specimen A	AS1–AP1	D1 (50 mm × 100 mm)
	AS3–AP3	D2 (100 mm × 100 mm)
	AS5–AP5	D3 (150 mm × 100 mm)
Specimen B	BS1–BP1	D4 (100 mm × 50 mm)
	BS3–BP3	D5 (100 mm × 150 mm)

**Table 2:** Surface wave measurement amplitudes and abnormal values of specimen B.

Actuator Number	Group 1		Group 2		Group 3		Group 4		Group 5
	I	IX	II	VIII	III	VII	IV	VI	V
1	2.01	1.86	2.06	1.96	2.28	2.54	2.63	3.13	3.12
2	2.13	1.75	2.35	2.16	2.46	2.62	2.54	2.51	3.35
3	1.94	1.78	2.31	2.08	2.19	2.39	3.06	2.78	2.87
4	1.89	1.93	2.18	2.04	2.56	2.33	2.96	2.76	3.15
5	2.17	2.14	1.96	2.35	2.43	2.45	2.44	2.97	3.06
6	1.96	2.07	2.08	2.09	2.55	2.27	2.86	2.66	3.34
7	2.03	1.88	2.33	2.37	2.38	2.89	2.76	3.01	3.11
8	1.74	**4.02**	1.93	**4.33**	2.75	2.76	3.12	3.16	3.26
9	1.85	2.01	2.46	2.12	2.69	**4.71**	3.08	2.86	3.19
10	1.93	1.74	2.34	2.24	2.54	2.31	2.78	2.67	**5.71**
11	2.11	1.66	**4.28**	1.98	**4.66**	2.65	2.96	2.94	3.29
12	**3.96**	1.87	2.41	2.47	2.46	2.37	3.21	2.48	3.38
13	1.84	2.16	2.29	2.23	2.72	2.86	2.66	2.78	2.78
14	1.86	**3.94**	2.08	**4.51**	2.61	2.77	2.93	3.23	2.91
15	1.97	1.88	2.17	**4.46**	2.42	**4.91**	3.16	**5.44**	3.13
16	2.13	1.96	2.32	2.19	2.67	2.84	**5.25**	**5.16**	**5.84**
17	1.98	1.62	**4.39**	2.14	**4.83**	2.45	**5.37**	3.07	3.38
18	**3.89**	1.79	**4.41**	1.89	2.35	2.33	3.05	3.24	3.41
19	2.06	2.04	2.44	2.36	2.47	2.24	3.14	3.21	3.06
**J** **udgment value**	**2.69**		**3.03**		**3.37**		**3.84**		**4.34**

## Data Availability

The original contributions presented in this study are included in the article. Further inquiries can be directed to the corresponding author.
